# Herlyn-Werner-Wunderlich syndrome: Diagnosis and treatment of an atypical case and review of literature

**DOI:** 10.1016/j.ijscr.2019.08.035

**Published:** 2019-09-13

**Authors:** Camila Girardi Fachin, João Lucas Aleixes Sampaio Rocha, Amanda Atuati Maltoni, Raquel Lins das Chagas Lima, Vitória Arias Zendim, Miguel Angelo Agulham, Alina Tsouristakis, André Ivan Bradley dos Santos Dias

**Affiliations:** a Pediatric Surgery, Hospital de Clínicas, Federal University of Paraná, Curitiba, PR, Brazil; b Medical School, Federal University of Paraná, Curitiba, PR, Brazil; c Cooper Medical School of Rowan University, Camden, NJ, USA

**Keywords:** Herlyn-Werner-Wunderlich syndrome, Renal agenesis, OHVIRA syndrome, Case report, Hemi-hysterectomy, Müllerian ducts abnormalities

## Abstract

**Introduction:**

Herlyn-Werner-Wunderlich syndrome (HWWS), also known as OHVIRA syndrome, is a rare congenital abnormality of Müllerian duct development characterized by uterus didelphys, unilateral blind hemivagina, and ipsilateral renal agenesis.

**Presentation of case:**

In this report, the authors present the case of a 12-year-old patient presenting with progressive and disabling abdominal pain after the onset of menarche diagnosed with HWWS, describe the various diagnostic modalities and treatment options available, along with a current review of the literature.

**Discussion:**

With normal external genitalia, HWWS is usually asymptomatic until menarche when patients present with worsening abdominal pain during menses and a palpable pelvic or abdominal mass. Untreated, HWWS may lead to a number of complications including endometriosis, infertility, and spontaneous abortion.

**Conclusion:**

Greater awareness of HWWS will lead to earlier detection and is the key to alleviating patient suffering and avoiding potentially severe complications.

## Highlights

• In this report we present a rare case of HWWS diagnosed in a 12-year-old female presenting with disabling abdominal pain after the onset of menarche.
• We reviewed patient’s clinical history, utility of diagnostic modalities, usual treatments, and recent literature.
• The purpose of this case report is to offer a better understanding of the pathophysiology of HWWS.
• When a young female presents with common, nonspecific symptoms, HWWS should be on differential diagnoses list.
• Greater awareness of HWWS will lead to earlier detection and, consequently, reduced complications caused by delayed diagnosis.

## 1 Introduction

Herlyn-Werner-Wunderlich syndrome (HWWS), also known as OHVIRA syndrome, is a rare congenital defect in the formation of Müllerian ducts, the developmental precursors to the fallopian tubes, uterus, cervix, and upper vagina [1]. Anatomically, HWWS is characterized by three key anomalies of the female reproductive tract: uterus didelphys, unilateral blind hemivagina, and ipsilateral renal agenesis. With normal external genitalia, diagnosis is often delayed until after menarche [3] with most affected patients presenting with progressive dysmenorrhea and a suprapubic mass on abdominal exam. Other common presenting symptoms include intermenstrual bleeding, mucopurulent vaginal discharge, and fever [[2], [3], [4]]. Early diagnosis is key to preventing complications such as endometriosis, infertility, and spontaneous abortion.

In this report we present a rare case of HWWS diagnosed in a 12-year-old female presenting with progressive and disabling abdominal pain after the onset of menarche. We recount the patient’s clinical history, illustrate the utility of various diagnostic modalities, describe common treatments, and review recent literature. The purpose of this case report is to offer a better understanding of the pathophysiology of HWWS so that when a young female presents with common, nonspecific symptoms, treating physicians may include HWWS on their list of differential diagnoses. Greater awareness of HWWS will lead to earlier detection and, consequently, reduced complications caused by delayed diagnosis. Written informed consent was obtained from the patient and from her parents for publication of this case report and accompanying images. A copy of the written consent is available for review by the Editor-in-Chief of this journal on request. This work has been reported in line with the SCARE criteria [5].

## 2 Case report

A 12-year-old female was referred to the University hospital with incapacitating dysmenorrhea, nausea, vomiting, fever, and a palpable abdominal mass in the hypogastric region. The patient described her symptoms as an acute exacerbation of a more chronic history of similar episodes that initially presented soon after the onset of menses at age 11. At first symptoms were relatively mild but they increased in intensity with each subsequent menstrual cycle. The patient reported that since menarche, her menstrual cycles have been irregular with moderate bleeding lasting an average of 5 days.

**Fig. 1:**
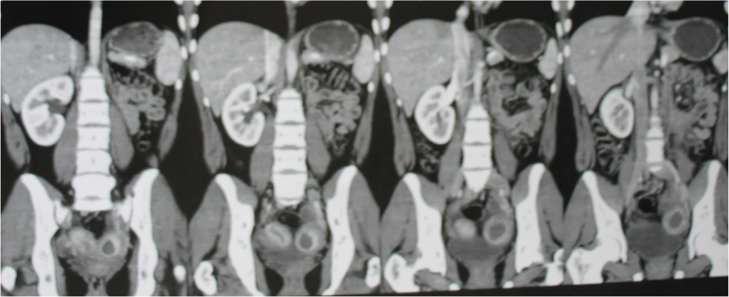
CT demonstrating absent left kidney, uterine duplication, suggestive of cervical duplication, and distension of the left cavity by hypodense material.

**Fig. 2:**
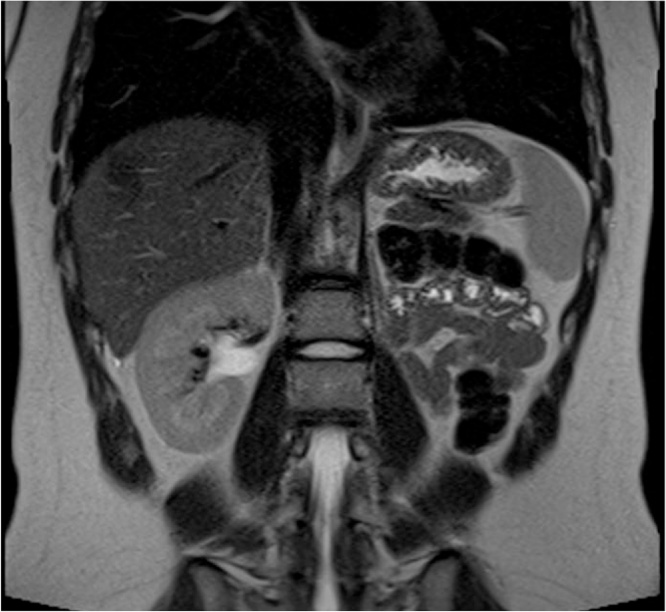
Abdominal MRI, coronal view, showing the absence of the left kidney.

**Fig. 3:**
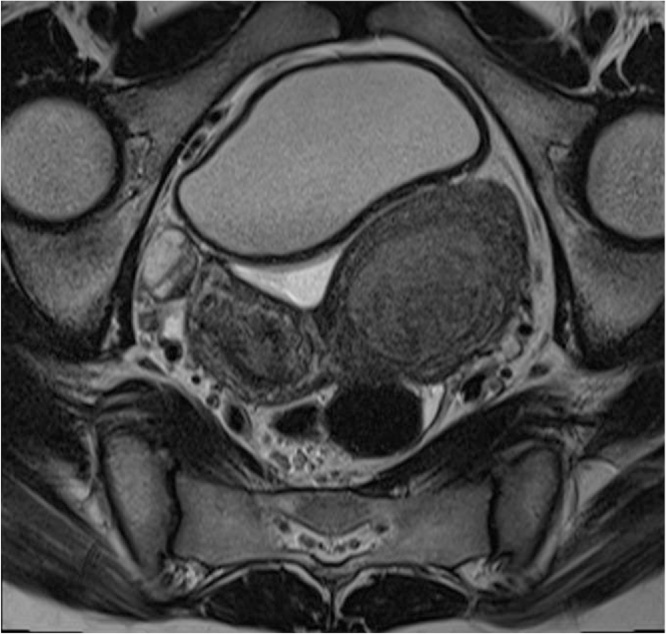
Pelvic MRI, transverse T2-weighted image, depicting a duplicated uterus and the presence of two endometrial cavities: right-sided cavity with 3.8 mm thickness and distended left cavity filled with hyperintense material.

Upon admission, the patient was prescribed levonorgestrel 0.15 mg and etilnilestradiol 0.30 mg to induce amenorrhea and attenuate her symptoms, however, the medications had no effect. A pelvic ultrasound revealed bifurcation of the uterine body near the uterine cervix, suggesting uterus bicornis. In addition to the ultrasound, computed tomography (CT) and magnetic resonance imaging (MRI) were performed (Fig. 1, Fig. 2, Fig. 3), both demonstrating uterus didelphys, left renal agenesis, and the presence of two endometrial cavities: a right-sided cavity with a thickness of 3.8 mm and a left-sided cavity filled with a hypodense material, likely demonstrative of blood.

**Fig. 4:**
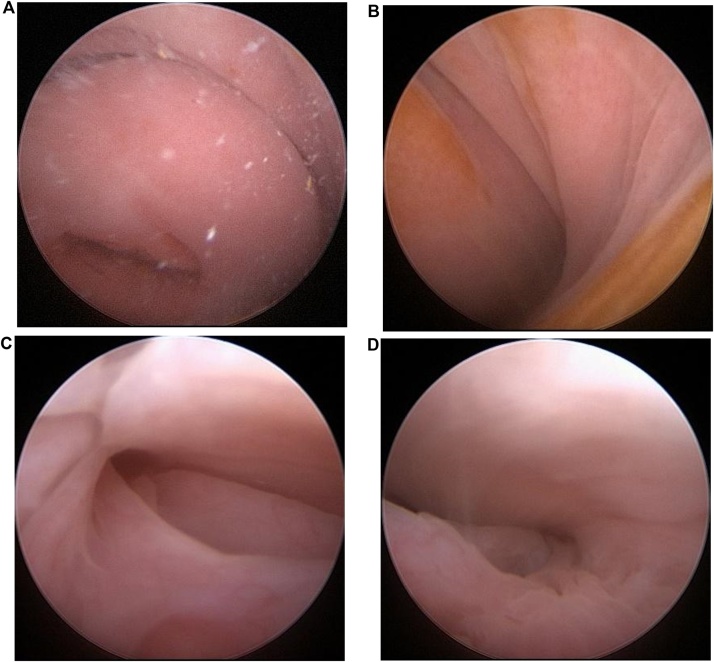
Images obtained via hysteroscopy. A) Single and normal cervix on the right. B) Absence of bulging on the left lateral wall of the vagina (dashed line) or its recess. C) Ostium of the right uterine tube (dashed circle), without alterations. D) Absence of ostium of the left uterine tube.

Under the diagnostic suspicion of Herlyn-Werner-Wunderlich Syndrome, the patient was referred to the University hospital. Initially, injectable medroxyprogesterone acetate was administered to block the hypothalamic-gonadal axis and interrupt the menstrual cycle. A colpo-hysteroscopy was then performed to confirm the clinical hypothesis and to determine the presence of a communication between the patent hemivagina and the atretic hemivagina. Hysteroscopic examination revealed a right-sided patent hemiuterus with a single uterine cervix, uterine tube, and ostium and a left-sided atretic hemiuterus without any lateral vaginal wall bulging or associated left uterine tube ostium (Fig. 4). The medical team did not have a clinical suspicion of cervical atresia of the left system prior to hysteroscopy, this diagnosis was made during the procedure. It changed the approach because if the left system wasn’t atretic and if there were a hemivagina with a septum, the septum could have been opened during hysteroscopy and no further treatment would be necessary. Since there was no left cervix and no possibility to drain the left system, hemi-histerectomy was performed. Since there was a huge hidrosalpingis, the medical team chose to remove the left tube with the left hemiuterus, and not only communicate the myometrium between the two uterine segments, because the left system was no longer functional.

**Fig. 5:**
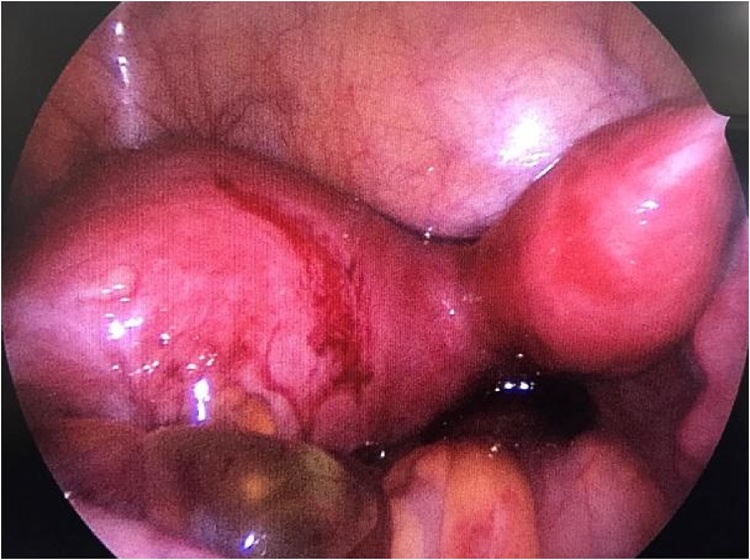
Laparoscopic hysteroscopy. Visualization of the larger left hemiuterus (left side of image) and right hemiuterus of smaller volume. Note the myometrial bridge connecting the two structures.

**Fig. 6:**
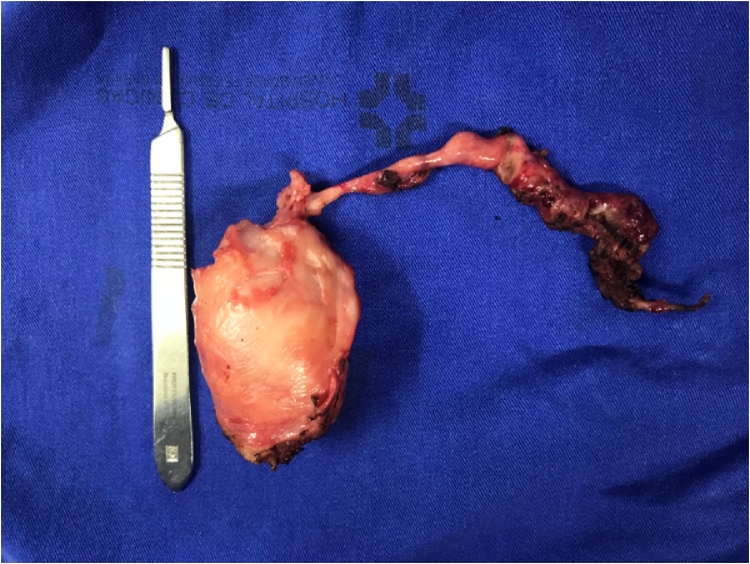
Left hemiuterus and uterine tube after laparoscopic resection.

In this approach, it was possible to observe a dilated left uterine tube, firmly adhered to the left ovary, which had an inflammatory aspect and a small hemorrhagic cyst. Uterus bicornis was identified, and the left hemiuterus was distended and connected to the right one only by a myometrial strand (Fig. 5). A left salpingectomy was performed with preservation of the left ovary, followed by left hemi-hysterectomy, ligation of the left uterine vessels, and resection of the myometrial segment connecting the two hemiuteri at a level adjacent to the cervix of the right hemiuterus (Fig. 6). Upon its resection, it was observed that the left hemiuterus had no communication to either the right-sided hemiuterus or right-sided vagina.

One day after the laparoscopic hemi-hysterectomy, the patient was discharged from the hospital. On follow-up, 1 month postoperatively, she had no complaints. One year after surgery, she remains asymptomatic.

## 3 Discussion

HWWS is one of many manifestations of Müllerian ducts abnormalities (MDA), which have an estimated incidence of 0.1%–3.8% [6]. The American Fertility Society classifies MDAs based on the stage of embryogenesis in which the defects occurred: complications in Müllerian organogenesis may lead to uterine hypoplasia, agenesis, or uterus unicornis; defective fusion of Müllerian ducts may result in either uterus bicornis (2 horns and 1 cervix) or uterus didelphys (2 horns and 2 cervix); and insufficient reabsorption of the uterine septum may cause a septated or arched uterus [7].

Of all MDAs, HWWS is a rare variant that occurs in only 5% of affected individuals [8]. While uterus didelphys is the most common feature of HWWS, there is a much wider variety of anatomical presentations possible. While the syndrome is more commonly diagnosed in puberty, with the presentation of dysmenorrhea soon after menarche [3], cases of neonatal and late presentations have also been reported [13,14].

Magnetic resonance imaging (MRI) is the imaging modality of choice for the diagnosis and classification of MDA as it provides details about uterine morphology, including contour and intrauterine cavity shape and continuity with each vaginal lumen, and nature of the fluids in these cavities. It can also identify associated pathologies such as endometriosis, pelvic inflammation and adhesions, as well as renal abnormalities [17]. In the case reported here, CT was requested by a urologist before HWWS was suspected, however, it is important to note that CT imaging is not recommended for syndrome diagnosis as it is less accurate and subjects the patient to ionizing radiation [16]. If MRI is unavailable or if resultant images are inconclusive, diagnosis of HWWS may be confirmed, and in most cases may be treated, by colpo-hysteroscopy. Additionally, laparoscopy may also be performed [18].

**Fig. 7:**
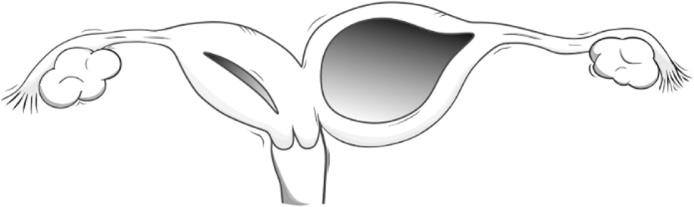
Schematic representation of the reproductive tract of the patient reported in this study. It is possible to note the existence of the two hemiuteri, connected to each other by a bridge of uterine tissue. The right hemiuterus is the only one containing a cervix, which communicates with a non-septate vagina.

After studying the characteristics of 79 patients with HWWS treated at the Peking Union Medical College Hospital (PUMCH), Zhu et al. proposed a new four-group classification system for the syndrome (Table 1) [2]. Unfortunately, the case presented in this report does not properly fit into this classification system. As schematically depicted in Fig. 7, our patient had only one patent hemivagina (right) that was in direct communication with the right cervix and right hemiuterus; on the left side, our patient had an atretic hemivagina with left cervical agenesis.

**Table 1:** Table created by the authors based on the classification system of HWWS proposed by Zhu et al. [2].

**Classification**	1		2	
**Subgroups**	1.1	1.2	2.1	2.2
**Anatomical Features**	Completely Obstructed Hemivagina		Incompletely Obstructed Hemivagina	
**Anatomical Features**	No communication between the two hemiuteri	No communication between the two hemiuterus	Hemiuterus isolated from each other	Duplicated cervix with a small communication between them
	Hemivagina containing a blind end	Cervicovaginal atresia	Communication between the two hemivaginas	No communication between the two hemivaginas

**Clinical Features**	**Symptom Onset & Diagnosis**			
	Early; Soon after menarche		Late; Years after menarche	
	**Dysmenorrhea**			
	Common		Less Common	
	**Intermittent Mucopurulent Discharge and Irregular Vaginal Bleeding**			
	Less Common		Common	
	**Hematometra, Hematosalpinx, Hematoperitoneum**			
	Common		Less Common	
	**Pelvic Inflamatory Disease**			
	Uncommon		Common	
	**Abdominal Pain, Fever, Emesis**			
	Common		Uncommon	
	**Progression to Secundary Endometriosis, Pelvic Adhesions, Pyosalpinx and Pyocolpos**			
	Quick, Easy		Gradual

Compared to patients with incomplete obstruction, patients with completely obstructed hemivagina are diagnosed earlier (often soon after menarche) and more likely to present with symptoms of abdominal pain, fever, and emesis than with mucopurulent vaginal discharge common in patients with incomplete obstruction. Additionally, women with complete obstruction are more susceptible to complications such as hematometra, hematosalpinx, hemoperitoneum, and even pelviperitonitis, as obstruction causes retention of menstrual flow in the internal female genitalia [10]. In the absence of treatment, the natural course of the disease also includes complications such as endometriosis and pelvic adhesions. While infertility and spontaneous abortions are also listed as common outcomes of untreated HWWS, studies have demonstrated that these outcomes are unlikely with proper treatment. A retrospective study of 36 patients with HWWS over a 30-year period revealed that after treatment 87% of patients with a desire to become pregnant had a successful pregnancy, with a total rate of 77% live births (15% premature, 62% at term) [12].

Surgery is important to relieve the obstruction, alleviate symptoms, and prevent complications of retrograde flow. Furthermore, close ultrasound follow-up and evaluation of kidney function is crucial in patients with HWWS due to the syndrome’s associated ipsilateral renal agenesis [10].

In a review of 87 cases, Fedele et al. described an anatomical variation of the didelphic uterus with cervical atresia [11]. The case here reported, despite the similarity of clinical presentation with that pattern, presents a subtle anatomical difference that implies a modification of the therapeutic approach. The situation described by Fedele et al. demands video-assisted cervicoplasty for the opening of the cervix and drainage into the vagina. The patient we describe in this report presented had left-sided cervical agenesis with a small myometrial bridge but had no communication with the hemivagina, which not only made a transvaginal therapeutic approach impossible, but also prevented preservation of the hemiuterus, which was ultimately removed laparoscopically.

It should be noted that in the majority of cases, the surgery of choice is the resection of the vaginal septum via colpohysteroscopy with preservation of the hemiuterus [2], but in this case this approach would have been ineffective, since the uterine cervix itself was absent. The surgery performed on our patient was a laparoscopic hemi-hysterectomy with salpingectomy. The most notable intraoperative finding was the complete absence of communication between the resected hemiuterus and both the contralateral hemiuterus and with the vagina.

We emphasize that, despite the fact that the symptomatology presented by patients is often nearly identical to that described in the literature, diagnosis is almost always delayed. In general, HWWS should be suspected in adolescents with menstrual changes, cyclic pelvic pain, renal agenesis, and abdominal or pelvic masses. Other less common but noteworthy symptoms include urinary retention by hematocolpic compression, recurrent urinary tract infections, and pyocolpos (secondary infection of the blood retained in the obstructed hemivagina) [15,16].

HWWS is a rare disorder that is relatively unknown by medical professionals and its associated dysmenorrhea is often misdiagnosed as other, more common causes of dysmenorrhea in teenagers. Additionally, diagnosis is further hindered by the administration of medications in order to relieve the dysmenorrhea in affected patients. The use of anti-inflammatory drugs and oral contraceptives, for example, change pain patterns and reduce, or even eliminate, menstrual flow, leading to symptom suppression and delay the correct and timely recognition of the disease [9].

## 4 Conclusion

It is imperative that physicians, especially pediatricians and gynecologists, develop strong awareness and keen clinical suspicion of HWWS when encountering patients with the seemingly common, nonspecific symptoms characteristic of the syndrome. Knowledge of the pathophysiology of HWWS and the syndrome’s clinical presentation is the basis for correct diagnosis and timely treatment is the key to alleviating patient suffering and avoiding potentially severe complications.

## Funding

This research received no specific grant form any funding agency in the public, commercial or not-for-profit sectors.

## Ethical approval

This project has been approved in the ethics committee: Comitê de Ética em Pesquisa Complexo Hospital de Clínicas UFPR –Curitiba, Brazil (Committee on Research Ethics from the Complex Clinic’s Hospital of the Federal University of Paraná – Curitiba – Brazil) on 5 March 2019. Project Number: 07695019.4.0000.0096.

## Consent

Written informed consent was obtained from the patient and from the patient's guardian for publication of this case report and all accompanying images.

## Author contribution

**Camila Girardi Fachin**: Conceptualization; Investigation; Project Administration

**Amanda Atuati Maltoni**: Writing – Original Draft

**João Lucas Aleixes Sampaio Rocha**: Writing – Original Draft

**Raquel Lins das Chagas Lima**: Writing – Original Draft

**Vitória Arias Zendim**: Writing – Original Draft

**Alina I. Tsouristakis**: Writing – Review & Editing

**Miguel Angelo Agulham**: Supervision

**André Ivan Bradley dos Santos Dias**: Conceptualization; Investigation; Project Administration.

## Registration of research studies

This is a case report only, not a clinical trial or research.

## Guarantor

Camila Girardi Fachin.

## Provenance and peer review

Not commissioned, externally peer-reviewed.

## Declaration of Competing Interest

The authors have none conflicts of interests to declare.
